# Temporal separation of two fin whale call types across the eastern North Pacific

**DOI:** 10.1007/s00227-012-2061-z

**Published:** 2012-09-14

**Authors:** Ana Širović, Lauren N. Williams, Sara M. Kerosky, Sean M. Wiggins, John A. Hildebrand

**Affiliations:** Scripps Institution of Oceanography, University of California San Diego, La Jolla, CA 92093-0205 USA

## Abstract

Fin whales (*Balaenoptera physalus*) produce a variety of low-frequency, short-duration, frequency-modulated calls. The differences in temporal patterns between two fin whale call types are described from long-term passive acoustic data collected intermittently between 2005 and 2011 at three locations across the eastern North Pacific: the Bering Sea, off Southern California, and in Canal de Ballenas in the northern Gulf of California. Fin whale calls were detected at all sites year-round, during all periods with recordings. At all three locations, 40-Hz calls peaked in June, preceding a peak in 20-Hz calls by 3–5 months. Monitoring both call types may provide a more accurate insight into the seasonal presence of fin whales across the eastern North Pacific than can be obtained from a single call type. The 40-Hz call may be associated with a foraging function, and temporal separation between 40- and 20-Hz calls may indicate the separation between predominately feeding behavior and other social interactions.

## Introduction

Seasonal sound production has been studied extensively in terrestrial animals, bird songs in particular (Catchpole and Slater 2003), but seasonal patterns in call production are also common among fishes and marine mammals. Generally, seasonal sound production is related to biologically meaningful behaviors such as reproduction, mate attraction, and foraging. For example, sciaenid fishes produce more sounds during peak spawning season than at other times of the year (Aalbers 2008; Locascio and Mann 2011; Ramcharitar et al. 2011). Among marine mammals, pinnipeds exhibit a seasonal cycle in their breeding calls (Thomas and Demaster 1982; Stirling et al. 1983), and cetaceans such as humpback (*Megaptera novaeangliae*), blue (*Balaenoptera musculus*), and fin whale (*B. physalus*) males sing most commonly during and around their presumed breeding season in the winter (Winn and Winn 1978; Mattila et al. 1987; Watkins et al. 1987; McDonald et al. 2001; Croll et al. 2002; Oleson et al. 2007a). In the late spring and during the summer, on the other hand, both sexes of these species produce distinct sounds associated with feeding (Thompson et al. 1986; Oleson et al. 2007a). In addition to seasonal patterns, daily patterns in sound production have been documented for fishes and marine mammals, and they may inform on the behavioral context of each sound. Night chorusing of sciaenids corresponds to the peak daily occurrence of spawning events (Holt et al. 1985). Many odontocetes, such as dolphins and porpoises, produce a majority of their echolocation signals during the night, this sound production corresponding to periods of foraging on vertically migrating prey (Carlström 2005; Johnston et al. 2008; Soldevilla et al. 2010). Blue whales, on the other hand, feed and produce foraging-associated calls mostly during the day, while their song has a crepuscular pattern (Stafford et al. 2005; Wiggins et al. 2005; Oleson et al. 2007a). Thus, studying temporal patterns in sound production can provide insight into both the behavioral ecology of a species, and its seasonal distribution.

Fin whales produce a variety of low-frequency (mostly <100 Hz), high-intensity (up to 189 dB re: 1 μPa at 1 m), short-duration (approximately 1 s), frequency-modulated sounds (Watkins 1981; Watkins et al. 1987; Edds 1988; Širović et al. 2007). The most often reported fin whale sound is the “20 Hz pulse,” a short-frequency downsweep mostly centered around 20 Hz, which is produced by fin whales worldwide (Watkins 1981; Edds 1988; Thompson et al. 1992; Clark and Fristrup 1997; Clark and Charif 1998; Watkins et al. 2000; Nieukirk et al. 2004; Širović et al. 2004; Castellote et al. 2012). This call is produced in regular sequences forming stereotyped songs, sometimes in a form of “doublets”(Watkins et al. 1987; McDonald and Fox 1999) or as repeated individual pulses, with likely a reproductive function as they are produced only by males (Croll et al. 2002). When produced in irregular sequences or as call-counter calls, 20-Hz calls likely have a social purpose and may be used to maintain contact (McDonald et al. 1995; Edds-Walton 1997). One “higher frequency” call type reported by Watkins (1981) was also sweeping down in frequency, generally between 100 and 30 Hz, but most often from 75 to 40 Hz. This call was produced by fin whales mostly during the summer during deep dives, as individual or multiple calls, but never in a regular sequence (Watkins 1981). The remaining fin whale signals are more variable in character, and their social context is more poorly understood (Watkins 1981; Edds 1988).

The traditionally accepted and simplified view of baleen whale seasonal life history is that most species undergo a seasonal migration from higher latitude, summer feeding grounds to lower latitude winter calving grounds (Kellogg 1929). Based on a comprehensive analysis of historic whaling records, mark recovery tags, visual sightings, and acoustic recordings, Mizroch et al. (2009) concluded that fin whale seasonal distribution in the eastern North Pacific indicates a more complex movement pattern. At least some part of the population undergoes a seasonal migration between the Gulf of Alaska and US west coast feeding areas to the wintering areas off Baja California (Rice 1998; Mizroch et al. 2009). The Bering Sea generally has high concentrations of fin whales during the summer and may serve as a mixing ground for two distinct populations, eastern and western (Mizroch et al. 2009). Consistent with a seasonal presence of fin whales in the Bering Sea, near the Aleutian Islands, 20-Hz calls appear the most prevalent between May and August (Moore et al. 1998). Offshore recordings, however, do not show this seasonal pattern and between 55°N in the Gulf of Alaska to approximately 30°N off California, 20-Hz calls are detected most often between September and March (Moore et al. 1998; Watkins et al. 2000; Stafford et al. 2007) when the whales would be expected to be farther south based on the traditional migration theory. This may indicate that at least a part of the population remains year-round at productive higher latitudes locations, such as near Kodiak Island and in the Gulf of Alaska (Mizroch et al. 2009).

In addition to the hypothesized ocean-basin-wide migrating populations, there is evidence of resident fin whale populations in peripheral seas such as the Gulf of California, the East China Sea, and the Mediterranean (Tershy 1993; Canese et al. 2006; Mizroch et al. 2009), as well as off Southern California (Forney and Barlow 1998). Among these resident populations, fin whales are more abundant off Southern California in the summer (Forney and Barlow 1998), while in Canal de Ballenas, the area with the highest fin whale abundance in the northern Gulf of California, their peak presence is in the winter and spring, with fewer sightings during the summer (Tershy 1993). It is interesting, however, that peak acoustic presence based on 20-Hz calls off Southern California lags the peak in presence from visual survey and occurs in the fall and winter (Clark and Fristrup 1997; Oleson 2005). These inconsistencies between visual and acoustic data indicate that a more thorough inspection of fin whale calling repertoire is needed to fully describe their seasonal presence.

In this paper, we describe the differences in seasonal and daily temporal patterns between the 20-Hz call and a higher frequency sound we designated the 40-Hz call after Watkins (1981), produced by fin whales at three locations across the eastern North Pacific Ocean: the Bering Sea, Southern California, and the Gulf of California (Canal de Ballenas). We also discuss possible behavioral context of these calls and their importance for successful acoustic monitoring of fin whales across the eastern North Pacific.

## Methods

**Fig. 1:**
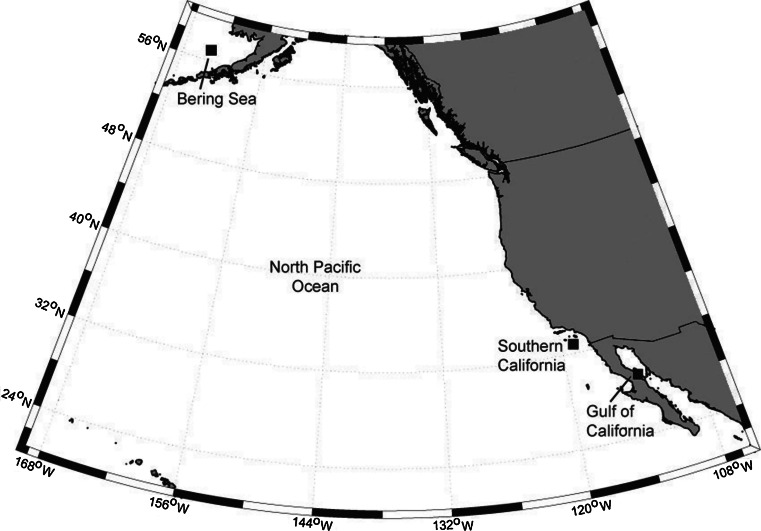
Map of the eastern North Pacific Ocean, with squares denoting locations where fin whale recordings were collected for this study: Bering Sea, Southern California, and Gulf of California

Passive acoustic data were collected using High-frequency Acoustic Recording Packages, HARPs (Wiggins and Hildebrand 2007), at three locations across the eastern North Pacific: the Bering Sea, off Southern California, and in the northern Gulf of California (Fig. 1). The HARPs recorded at different sample rates and duty cycles, but all the recordings provided sufficient bandwidth for capturing the full bandwidth of both fin whale call types (Table 1). Nine months of data (from April 2005 until January 2006) were available in the Bering Sea, a full year from January 2010 through January 2011 was available for Southern California, and data were collected intermittently in the Gulf of California from August 2008 until May 2010. All recordings were decimated to reduce the data to 1 kHz effective bandwidth and allow for computationally faster analysis.

**Table 1:** Location, dates, and recording parameters for acoustic recordings at three locations across the eastern North Pacific

Location	Latitude (N)	Longitude (W)	Deployment period	Depth (m)	Sample rate (ksamples/s)	Duty cycle
Bering Sea	56° 51.6′	164° 03.5′	24 Apr 05–16 Jan 06	70	40	Continuous
Southern California	32° 22.2′	118° 33.8′	31 Jan 10–26 Mar 10	1280	200	Continuous
			11 Apr 10–18 Jul 10			
			23 Jul 10–8 Nov 10			
			7 Dec 10–30 Jan 11			
Gulf of California	29° 01.6′	113° 22.5′	8 Aug 08–25 Dec 08	690	200	5/20
			24 Apr 09–12 Sep 09			5/15
			7 Dec 09–18 May 10			5/15

For duty cycle of the recording, the first number denotes the length of recording [min] during each cycle, and the second number is the total length [min] of each cycle

As there are no previous detailed characterizations of the 40-Hz call type, we measured start and end frequency of 40-Hz calls at all three location. We performed measurements of 20 calls at each location, with each call separated by at least 24 h to try to ensure calls from a single animal are not overrepresented in the data. We tested whether call frequency characteristics at different locations were similar enough to each other to be considered as coming from the same population using a one-way analysis of variance. The start and end frequency characteristics of the 40-Hz calls did not vary among locations (ANOVA, *F*_(2,57)_ = 0.04, *p* = 0.964 and *F*_(2,57)_ = 0.79, *p* = 0.461, respectively), so we pooled all the measurements for the calculation of overall average frequency characteristics.

Long-term spectral averages (LTSAs), with 5 s temporal and 1 Hz frequency resolution, were calculated from the data using the custom MATLAB-based program *Triton* (Wiggins and Hildebrand 2007). These long-term averages were manually scrutinized by trained analysts for the presence of 20- and 40-Hz calls in each hour of data. The analysts visually examined 1 h LTSA windows. If there was uncertainty regarding the call type based on the LTSA, the analysts would further examine the call with a shorter time scale (20 s to 2 min) spectrogram with higher temporal resolution (0.1 s) than the LTSAs to verify the call type based on spectral characteristics and durations of individual calls. Since hourly presence of calls was noted rather than the timing of individual calls, we did not further classify 20-Hz calls into songs or call-counter call. Fin whales are known to produce 20-Hz calls in regular sequences, while 40-Hz calls can be produced repetitively but generally not in a regular sequence (Watkins 1981). Thus, the presence of calls in an hour of data was used as a metric to remove some of the positive bias to the 20-Hz calls that would result from their regular repetitiveness had we used individual call detections, although a bias remains in the increased likelihood of detection of long sequences of calls over individual or irregular calls. Additionally, the Gulf of California data were recorded using a duty cycle, making it more difficult to measure the repetitiveness of calls to further classify 20-Hz calls. Percent of hours with calls per week was plotted for each call type and each location. Less than 100 % effort in data collection per week, either as a result of deployment or recovery of instruments or duty-cycled data, was plotted along with call data. The Gulf of California data are presented on a 1-year timeline to facilitate observation of seasonal patterns even though the data were collected over the course of 3 years. To obtain a quasi-continuous year-long time series, we plotted the following sections of data from each deployment: September 1–December 15, 2008, May 1–August 31, 2009, and December 16, 2009–April 31, 2010.

We calculated the yearly mean day of calling for each call type at each location using circular statistics toolbox for MATLAB (Berens 2009). The measured variable in this analysis was the day when each hour with a call was recorded, and thus, each day was assigned an angular value based on its position on an imaginary annual circle. The mean day of calling, with its associated 95 % confidence interval, was calculated from the angular values. To test whether the mean days of calling for 40- and 20-Hz calls differed significantly at each location, we performed a parametric Watson–Williams multi-sample test for equal means (Zar 1984). This analysis was performed on the same data that were used for creating seasonal plots for each site.

To test if calls were preferentially produced by fin whales at a certain time of the day, we divided the hours with detected calls into 4-day periods: dawn, day, dusk, and night. Dawn was defined as the hours of nautical twilight (defined by the passage of the center of the sun geometrically 12° below the horizon) start and sunrise, as well as any hours between them. Dusk consisted of the hours from sunset to nautical twilight end. Day was made up of hours after sunrise but before sunset, and night were hours after the end but before the start of nautical twilight. This method overestimates total period of dawn and dusk and underestimates day and night, because the entire hour at change of sun condition is considered as dawn or dusk, regardless of the exact time of change. Sunrise, sunset, and twilight information were accessed from the US Naval Observatory sun and moon rise/set tables (http://aa.usno.navy.mil/data/docs/RS_OneYear.php). The total number of hours with calls was summed up for each day period over the entire duration of the recordings. We used chi-square goodness of fit to test the hypothesis that the number of hourly detections of each call for each day period at each site (observed value) is proportional to the total effort hours for that day period (expected value). We used α = 0.05 to reject the null hypothesis that whales do not call preferentially during any day period. To graphically represent the variability in diel patterns, we plotted the values as a difference between the actual and expected call presence values, expressed as a fraction of total effort.

## Results

**Fig. 2:**
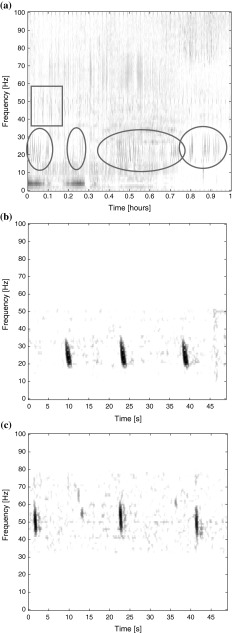
**a** Long-term spectral average plot (2000-point fast Fourier transform (*FFT*), 5 s time average and 2 kHz sample rate) recorded on June 16, 2010 with 20-Hz calls marked with *ovals* and 40-Hz calls marked with a *rectangle*. Spectrograms of fin whale (**b**) 20-Hz calls (2000-point FFT with 95 % overlap, *band*-pass filtered between 10 and 50 Hz; sample rate 2 kHz) recorded on June 9, 2010 and **c** 40-Hz calls (2000-point FFT with 95 % overlap, *band*-pass filtered between 30 and 80 Hz; sample rate 2 kHz) recorded on April 18, 2010. All recordings were collected off Southern California

Two fin whale call types recorded in the eastern North Pacific, the 20-Hz and the 40-Hz call, were both short-duration downsweeps, but with different frequency characteristics (Fig. 2). The 40-Hz calls downswept from 61.2 ± 6.6 to 47.6 ± 5.7 Hz (*N* = 60) over about 1 s. The calls were generally not produced in regularly repeated sequences.

**Fig. 3:**
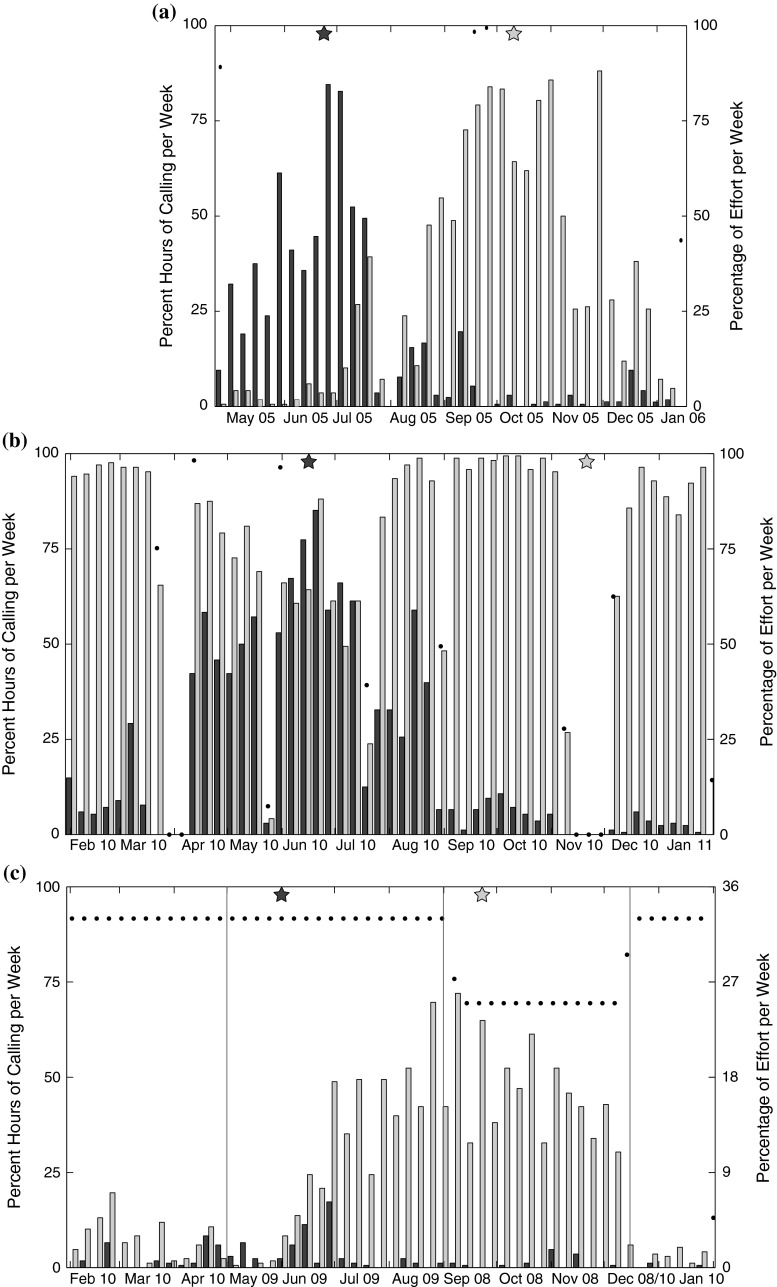
Percent of hours per week with fin whale 20-Hz (*light gray*) and 40-Hz (*dark gray*) calls recorded **a** in the Bering Sea, **b** off Southern California, and **c** in the Gulf of California. *Right axes* and *black dots* represent percentage of recording effort per week when effort was less than 100 %. Note that in the Gulf of California (**c**), the scale of the *right axes* is different because the recordings were on a duty cycle. Also note that the data in *panel* (**c**) are not continuous and *vertical lines* denote times when data from different deployments were used. *Stars* at the *top* of each *panel* represent the mean day of calling presence for 20-Hz (*light gray*) and 40-Hz (*dark gray*) calls during the year. All *panels* are aligned by month, for easier seasonal comparison

Both the 20 and 40 Hz fin whale calls were recorded at all three locations. Additionally, they were present during all months with recordings although the relative occurrence of their weekly presence varied within and among sites (Fig. 3). For example, the 40-Hz call was least common in the Gulf of California, where a maximum of 17 % of hours per week had calls. If corrected for the recording duty cycle, the rate was still the lowest at 51 % of hours per week with calls. Off Southern California and in the Bering Sea, on the other hand, a maximum of about 85 % of hours had 40-Hz calls during times of peak presence. The peak in 40-Hz calling occurred in June at all three locations, albeit the earliest in the Gulf of California and the latest in the Bering Sea (Table 2). The 20-Hz call was the most common off Southern California with nearly 100 % of hours with calls during large parts of the year, but with a notable decrease from May until July (Fig. 3). The presence of this call was more seasonal in the Bering Sea and the Gulf of California, with a high number of hours with calls occurring between July and December. The peak in 20-Hz calling was more variable than the peak in 40-Hz calls, and it occurred the earliest in the Gulf of California and the latest off Southern California (Table 2). The difference between the yearly mean calling day between the two call types was significant in the Bering Sea and off Southern California (Watson–Williams test, *F* = 27.1, *p* < 0.001 and *F* = 936.3, *p* < 0.001, respectively), but the means were not significantly different in the Gulf of California (Watson–Williams test, *F* = 0.42, *p* = 0.516).

**Table 2:** Yearly mean day of calling presence (and 95 % confidence interval) for each call type at each of the three locations across the eastern North Pacific

	Gulf of California	Southern California	Bering Sea
40-Hz call	1 June (21 May–13 June) *N* = 169	15 June (13–18 June) *N* = 1,901	25 June (23–29 June) *N* = 1,136
20-Hz call	23 September (20–27 September) *N* = 2,319	21 November (25 October–18 December) *N* = 6,436	8 October (6–11 October) *N* = 2,037

*N* is sample size used for each calculation, representing the number of hours with detected calls for each call type at each location

**Fig. 4:**
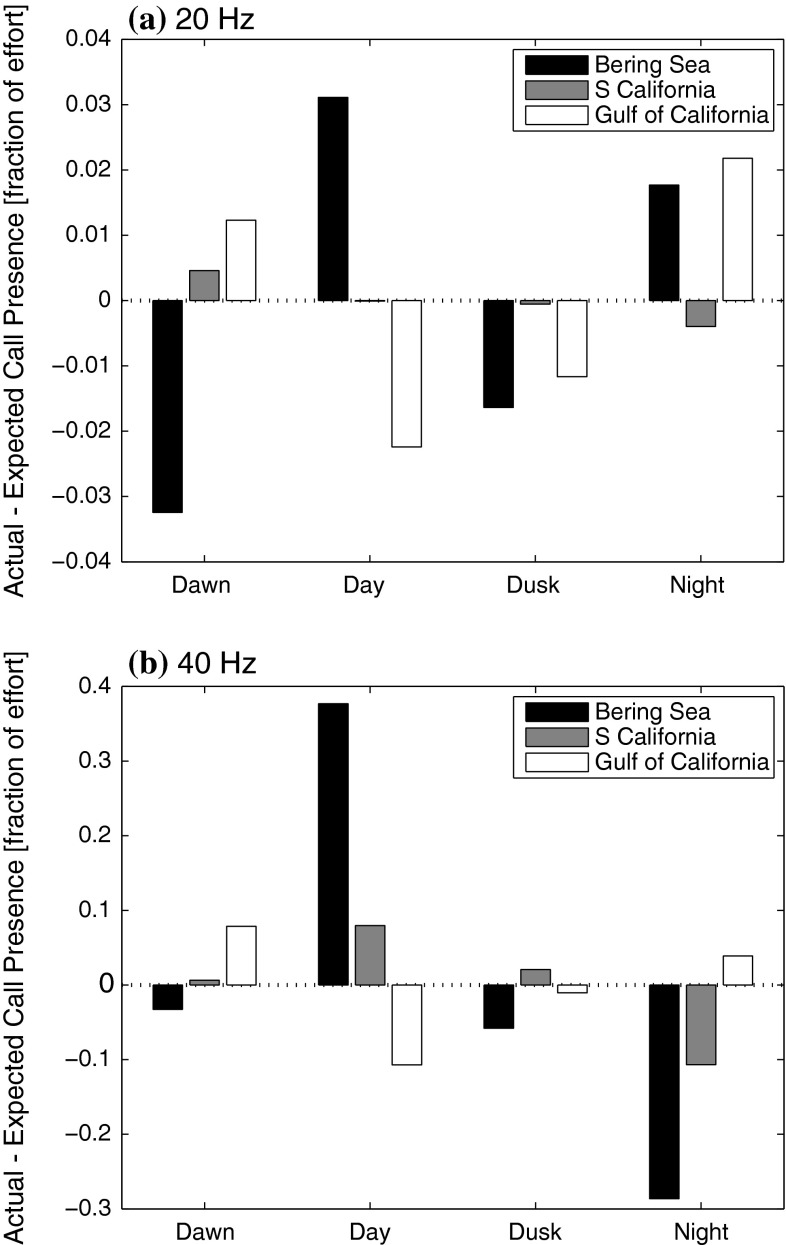
Difference between the actual and expected hourly call presence, in fractions of effort, for (**a**) 20-Hz and (**b**) 40-Hz call types during each of the 4 day periods: dawn, day, dusk, and night. *Black bars* represent data from the Bering Sea, *gray* are Southern California, and *white* are the Gulf of California data

Neither the 20- nor 40-Hz calls were produced uniformly during the day across the eastern North Pacific (Fig. 4). The diel calling pattern was consistent for the two call types within the site in the Bering Sea and in the Gulf of California, but the patterns between the calls differed off Southern California. In the Bering Sea, both 20- and 40-Hz calls were more common during day hours and less common during dusk and dawn (chi-square test, χ_3_^2^ = 29.86, *p* < 0.001 and χ_3_^2^ = 695.2, *p* < 0.001, respectively). This pattern was largely opposite in the Gulf of California. While the diel pattern was also significantly different from our null hypothesis, more hours with 40 and 20-Hz calls were detected during night and dawn (chi-square test, χ_3_^2^ = 20.20, *p* < 0.001 and χ_3_^2^ = 17.44, *p* = 0.001, respectively). Off Southern California hours with 40-Hz calls were more common during the day and dusk (chi-square test, χ_3_^2^ = 94.76, *p* < 0.001), while 20-Hz calls occurred less commonly during day and dusk hours, although the trend was not significantly different from the null hypothesis (chi-square test, χ_3_^2^ = 2.03, *p* = 0.567).

## Discussion

Both types of fin whale calls were detected at all three eastern North Pacific locations during all the months of recording and, combined, they provide a more accurate insight into the seasonal presence of fin whales across the eastern North Pacific than just a single call type. Fin whale calls were detected in the Bering Sea during the entire deployment period including the early winter. As we did not have data from February to April, we cannot conclude if fin whales are present in the Bering Sea during late winter. While there is a marked decrease in the number of hours with fin whale calls in December and January suggesting the whales may be leaving the area, fin whales have been recorded previously off Aleutian Islands in March and April (Moore et al. 1998), and it is possible some fin whales remain at these high latitudes year-round.

Year-round presence of both fin whale call types off Southern California and in the Gulf of California is consistent with previous reports of likely resident populations in those areas (Tershy et al. 1990; Forney and Barlow 1998). However, peak acoustic presence in the Canal de Ballenas was in the summer and fall, but fin whales are more commonly seen at this location during the winter and spring (Tershy et al. 1990). Off Southern California, on the other hand, overall calling presence was consistently high, while visual sightings peak occurs during the summer (Forney and Barlow 1998). These cases illustrate that visual and acoustic methods may result in different occurrence patterns (Širović et al. 2006; Gedamke and Robinson 2010) since each method depends on a broader behavioral context (e.g., visible surface behavior or subsurface sound production) and may change seasonally.

Production of 20 Hz pulses has been consistently attributed to fin whales (Schevill et al. 1964; Watkins and Schevill 1972), but the only published record attributing the 40-Hz call to fin whales comes from Watkins (1981). The exact locations of Watkins’ (1981) recordings were not noted though most of the recordings were made in the Atlantic Ocean. The frequency characteristics of 20-Hz calls produced by fin whales in the Atlantic and the Pacific Ocean are similar (Watkins 1981), and it is reasonable to assume 40-Hz calls recorded in the Pacific, that match Watkins’ (1981) description, are also produced by fin whales. In addition, the locations of our recordings offer further evidence that fin whales are the only species that is a likely source of this sound. We recorded these calls in the Bering Sea and the Gulf of California, both areas where fin whales are the most commonly sighted baleen whales (Tershy et al. 1990; Moore et al. 2002). Minke (*B. acutorostrata*) and humpback whales (*M. novaeangliae*), the other two baleen whale species common in the Bering Sea (Moore et al. 2002), are not commonly sighted in the northern Gulf of California (Tershy et al. 1990; Mangels and Gerrodette 1994). At the same time, the Bering Sea is well outside of the known range of Bryde’s whales (*B. edeni*), a tropical and subtropical species also common in the Gulf of California (Tershy et al. 1990). Additionally, sei whales (*B. borealis*) are very rarely sighted in the Bering Sea (Moore et al. 2002) and the Gulf of California (Mangels and Gerrodette 1994) and would thus be an improbable source of these common calls. Finally, ambiguity could arise in distinguishing blue whale D calls from fin whale 40-Hz calls in an LTSA even though D calls have a distinctly broader bandwidth (Oleson et al. 2007a). If bandwidth alone was not sufficient for classifying call type, a trained analyst would use a spectrogram with a higher time resolution and base classification on the different durations of the two call types (Oleson et al. 2007a). Since this additional step was conducted for hourly periods when there was ambiguity about the call type based on the LTSA, we conclude that the vast majority of hours with identified calls were indeed fin whale 40-Hz calls.

While both call types show consistent fin whale presence at these three eastern North Pacific locations, there were clear seasonal differences in the relative persistence of each call type during the period of our recordings. Fin whales produced 40-Hz calls primarily during the summer, with a peak in June, while peak in 20-Hz calling occurred in the fall (between late September and November). This seasonal variation could be an indication of the difference in the behavioral context during which each call type is produced. Watkins (1981) noted that higher frequency 40-Hz calls were generally produced by animals in groups, apparently in feeding contexts, for example when the animals were seen surface feeding and during deep, likely foraging dives (Croll et al. 2001). Fin whales are known to feed at all three sites; thus, their presence at these locations could be associated with feeding. The Bering Sea is an important feeding habitat where fin whales have been linked with concentrations of zooplankton and fish during the summer (Moore et al. 2000). In addition to the high latitude productive areas, fin whales in the eastern North Pacific also feed in productive regions off Southern California and in the Gulf of California, particularly in the Canal de Ballenas (Tershy 1992; Croll et al. 2001). The peak in calling presence of the 40-Hz call occurred in early summer at all locations, a pattern similar to blue whale D calls off Southern California (Oleson et al. 2007a), which have been associated with feeding whales (Oleson et al. 2007b). The blue whale is the most closely related animal to fin whale, so it is possible that call production could be similar in the two species. Based on the seasonal peak in the production of this call, its occurrence at locations known to be fin whale feeding areas, and association between behaviors associated with feeding and this call observed by Watkins (1981), we propose that the 40-Hz call may be produced by whales that are primarily focused on feeding, analogous to the downswept D call in blue whales.

There is wide agreement that 20-Hz calls, when occurring in regular, songlike sequences, likely serve a mating function, either as advertisement of resources (Croll et al. 2002) or for mate attraction (Watkins 1981; Watkins et al. 1987). Call-counter calls and irregular 20-Hz calls, on the other hand, likely serve social function and may be used for keeping contact with moving conspecifics (Edds 1988; McDonald et al. 1995), and, like 40-Hz calls, are generally produced by animals in groups (Watkins 1981). Nearly year-round high levels (>80 % of hours with calls from August until April) of 20-Hz calls off Southern California could be explained by the fact that we combined all variants of the 20-Hz call (song, counter call and irregular) into one metric in this study. From earlier studies, we know that call-counter calls occur year-round off British Columbia, but are seasonal off the US west coast (Moore et al. 1998) and in Hawaii (Thompson and Friedl 1982). Off Southern California, counter calls peak in the summer, while song phrases are only found in the winter (Oleson 2005). The later seasonal peak in the 20 Hz hourly call presence off Southern California, relative to the other locations, is likely the result of an extended fin whale presence off Southern California.

The persistence of a high hourly rate of 20-Hz calls for most of the year off Southern California may be a recent development. During the early 2000s, a clear peak in fin whale call detections off Southern California occurred in the fall with fewer 20-Hz calls detected in the winter (Oleson 2005). A possible explanation for this persistence of the 20-Hz call could lie in the changing oceanographic conditions. Over the last decade, there has been considerable fluctuation in the California Current ecosystem, driving fluctuations in abundance and distribution of lower trophic levels (Bjorkstedt et al. 2010). As a result of these oceanographic changes, postulated resident and migratory fin whale populations off Southern California (Mizroch et al. 2009) may be using these areas differently, contributing to more constant use levels. We see the same prolonged use of the area by fin whales in our recordings from the same location in the Southern California Bight a year earlier (February 2009–January 2010). The temporal separation of the two calls is maintained in the data, but there is a shift to earlier peaks, with the mean day of 40-Hz calls in late spring (6 May, 95 % confidence interval 30 April–12 May) and the mean day of 20-Hz calls in early winter (18 January, 95 % confidence 12–24 January). However, considering the greatly varying oceanographic conditions between these 2 years, with 2009 a strong El Niño and 2010 a moderate La Niña years (Lee et al. 2010), some variation in the seasonality can be expected. A better understanding of fin whale population structure followed by a detailed investigation into the variation in seasonal use of the different parts of the North Pacific by different fin whale stocks would be timely in the light of continued changes in the ocean environment driven by global climate change (Bakun 1990; Hayward 1997; King et al. 2011).

Unlike off Southern California, 20-Hz calls in the Bering Sea and the Gulf of California showed distinct seasonality, with peaks in the late summer and fall. These peaks are earlier than reported in most previous analysis of 20-Hz calls in the northeastern Pacific, where peaks were found to occur in the winter and early spring (Watkins et al. 1987; Watkins et al. 2000). Previous recordings in the Gulf of California, on the other hand, yielded predominately 20-Hz calls in March, some 20-Hz calls in August, and neither 20- nor 40-Hz calls in February or June (Cummings et al. 1986; Thompson et al. 1992). These studies provided only brief glimpses into fin whale acoustics in the Gulf of California, but we are not familiar with any year-round recordings from this area. While our data are non-contiguous and we may be confounding some intra-annual variability, we provide the first year-round acoustic look at fin whale presence in the Gulf of California. Some of the variability between our recordings and earlier reports could be due to the interannual variability inherent to the oceanographic environment as illustrated in our Southern California recordings. Additional complication to our interpretation stems from the fact that recordings from different basins are not temporally overlapping. However, we believe the overall seasonal pattern and separation of the two call types is likely to persist across years and regions (Moore et al. 1998, 2006; Watkins et al. 2000; Širović et al. 2004; Oleson et al. 2007a; Stafford et al. 2009).

In addition to seasonal differences, we also found evidence of differences in daily calling patterns across the eastern North Pacific for both call types. If calls are associated with different behaviors, a difference in diel patterns of calls is not surprising as daily patterns in fin whale behaviors have been documented worldwide. Feeding patterns vary between different oceans; in the North Atlantic fin whales feed during the night (Víkingsson 1997), while in the Southern California Bight and the Gulf of California they have been observed feeding during the day (Tershy 1992; Acevedo-Gutiérrez et al. 2002). Dominant production of the 40-Hz call in the Bering Sea and off Southern California occurred during the day with a decrease in the number of hours with calls at night. During the day, whales can forage more effectively on prey, such as euphausids, copepods, and schooling fishes (Kawamura 1980; Tershy 1992), which are aggregated at depth. This diurnal pattern supports our hypothesis that the whales may be producing these calls while foraging. The daily call production pattern is the opposite, however, in the Gulf of California, with peaks at night and dawn. Most observations of feeding in this area were from the winter and spring (Tershy 1992), but most calls occurred during the summer and fall, when fin whales in other areas are known to forage at night (Víkingsson 1997). It is possible that the whales change their feeding behavior during the year if the behavior of their prey or their prey preference changes. Although direct observations of fin whales during the night are difficult, in the Gulf of California, nighttime observations during the summer months would be needed to help explain the difference in the calling pattern.

While overall daily patterns in sound production were largely similar among sites, variation from expected number of hours with call was much smaller for 20-Hz calls, and in the case of Southern California was not even significant. Previous studies have found that a peak in 20-Hz calls in Southern California occurs after dusk (Oleson 2005), but it is not surprising that we found no significant diel pattern in 20-Hz calls off Southern California considering calls were detected close to 100 % of the time during long periods of the year. Additionally, a distinct diel pattern may exist in song and call-counter call, but combining the calls into one metric may obscure and confound any such patterns.

By augmenting the seasonal record of fin whale acoustic presence in the eastern North Pacific with the 40-Hz call, we provide evidence of consistent levels of fin whale presence in higher and mid-latitudes. When a call is detected, we know a whale is present, but absence of a call does not mean that whales are not present; they may be present but quiet. By combining multiple call types into the analyses of seasonal presence, we can diminish the bias of a quiet whale that may occur due to the behavioral constraints of calling and obtain seasonality information that more accurately reflects the animal’s true presence. Interestingly, the peak in 40-Hz calls corresponds better to whale presence based on visual data off Southern California, but neither call type offers good overlap with the peak abundance of whales based on visual data in the Gulf of California (Tershy et al. 1990; Breese and Terhsy 1993; Forney and Barlow 1998).

In conclusion, we have confirmed year-round presence of calling fin whales in the northern Gulf of California and off Southern California, as well as their presence in the Bering Sea from May through January. We propose that estimates of seasonality of fin whales would benefit from inclusion of both 20- and 40-Hz calls, as these calls are likely produced in distinct behavioral context, and thus, one may be favored over another during different times of the year. The 40-Hz call, likely associated with foraging, peaks in late spring (June) across the eastern North Pacific, while peaks in 20-Hz calling occur 3–5 months later. Direct studies of feeding and calling behaviors would help further explain factors driving the production of these calls, as well as their ultimate function.

## References

[CR1] Aalbers SA (2008). Seasonal, diel, and lunar spawning periodicities and associated sound production of white seabass (*Atractoscion nobilis*). Fish Bull.

[CR2] Acevedo-Gutiérrez A, Croll DA, Tershy BR (2002). High feeding costs limit dive time in the largest whales. J Exp Biol.

[CR3] Bakun A (1990). Global climate change and intensification of coastal ocean upwelling. Science.

[CR4] Berens P (2009) CircStat: a MATLAB toolbox for circular statistics. J Stat Softw 31:1–21

[CR5] Bjorkstedt E, Goericke R, McClatchie S, Weber E, Watson W, Lo N, Peterson B, Emmett B, Peterson J, Durazo R (2010). State of the California Current 2009–2010: regional variation persists through transition from La Niña to El Niño (and back?). Calif Coop Ocean Fish Invest Rep.

[CR6] Breese D, Terhsy BR (1993). Relative abundance of cetacea in the Canal de Ballenas, Gulf of California. Mar Mamm Sci.

[CR7] Canese S, Cardinali A, Fortuna CM, Giusti M, Lauriano G, Salvati E, Greco S (2006). The first identified winter feeding ground of fin whales (*Balaenoptera physalus*) in the Mediterranean Sea. J Mar Biol Ass UK.

[CR8] Carlström J (2005). Diel variation in echolocation behavior of wild harbor porpoises. Mar Mamm Sci.

[CR9] Castellote M, Clark CW, Lammers MO (2012). Fin whale (*Balaenoptera physalus*) population identity in the western Mediterranean Sea. Mar Mamm Sci.

[CR10] Catchpole CK, Slater PJB (2003). Bird song: biological themes and variations.

[CR11] Clark CW, Charif RA (1998) Acoustic monitoring of large whales to the west of Britain and Ireland using bottom-mounted hydrophone arrays, October 1996–September 1997. JNCC report no. 281, 281, Aberdeen, UK

[CR12] Clark CW, Fristrup KM (1997). Whales ‘95: a combined visual and acoustic survey of blue and fin whales off Southern California. Rep Int Whal Commn.

[CR13] Croll DA, Acevedo-Gutiérrez A, Tershy BR, Urbán-Ramírez J (2001). The diving behavior of blue and fin whales: is dive duration shorter than expected based on oxygen stores?. Comp Biochem Phys A.

[CR14] Croll DA, Clark CW, Acevedo A, Tershy BR, Flores S, Gedamke J, Urban J (2002). Only male fin whales sing loud songs. Nature.

[CR15] Cummings WC, Thompson PO, Ha SJ (1986). Sounds from Bryde, *Balaenoptera edeni*, and finback, *B. physalus*, whales in the Gulf of California. Fish Bull.

[CR16] Edds PL (1988). Characteristics of finback *Balaenoptera physalus* vocalizations in the St. Lawrence Estuary. Bioacoustics.

[CR17] Edds-Walton PL (1997). Acoustic communication signals of mysticete whales. Bioacoustics.

[CR18] Forney KA, Barlow J (1998). Seasonal patterns in the abundance and distribution of California cetaceans, 1991–1992. Mar Mamm Sci.

[CR19] Gedamke J, Robinson SM (2010). Acoustic survey for marine mammal occurrence and distribution off East Antarctica (30–80°E) in January–February 2006. Deep-Sea Res II.

[CR20] Hayward TL (1997). Pacific Ocean climate change: atmospheric forcing, ocean circulation and ecosystem response. Trends Ecol Evol.

[CR21] Holt GJ, Holt SA, Arnold CR (1985). Diel periodicity of spawning in sciaenids. Mar Ecol Prog Ser.

[CR22] Johnston DW, McDonald M, Polovina J, Domokos R, Wiggins S, Hildebrand J (2008). Temporal patterns in the acoustics signals of beaked whales at Cross Seamount. Biol Lett.

[CR23] Kawamura A (1980). A review of food of Balaenopterid whales. Sci Rep Whales Res Inst.

[CR24] Kellogg R (1929). What is known of the migrations of some of the whalebone whales. Smithson Inst Ann Rep.

[CR25] King JR, Agostini VN, Harvey CJ, McFarlane GA, Foreman MGG, Overland JE, Di Lorenzo E, Bond NA, Aydin KY (2011). Climate forcing and the California Current ecosystem. ICES J Mar Sci.

[CR26] Lee T, Hobbs WR, Willis JK, Halkides D, Fukumori I, Armstrong EM, Hayashi AK, Liu WT, Patzert W, Wang O (2010) Record warming in the South Pacific and western Antarctica associated with the strong central-Pacific El Nino in 2009–2010. Geophys Res Lett 37. doi:10.1029/2010gl044865

[CR27] Locascio JV, Mann DA (2011). Diel and seasonal timing of sound production by black drum (*Pogonias cromis*). Fish Bull.

[CR28] Mangels KF, Gerrodette T (1994). Report on cetacean sightings during a marine mammal survey in the Eastern Tropical Pacific Ocean aboard the NOAA ships McArthur and David Starr Jordan, July 28-November 6, 1993.

[CR29] Mattila DK, Guinee LN, Mayo CA (1987). Humpback whale songs on the North Atlantic feeding grounds. J Mammal.

[CR30] McDonald MA, Fox CG (1999). Passive acoustic methods applied to fin whale population density estimation. J Acoust Soc Am.

[CR31] McDonald MA, Hildebrand JA, Webb SC (1995). Blue and fin whales observed on a seafloor array in the Northeast Pacific. J Acoust Soc Am.

[CR32] McDonald MA, Calambokidis J, Teranishi AM, Hildebrand JA (2001). The acoustic calls of blue whales off California with gender data. J Acoust Soc Am.

[CR33] Mizroch SA, Rice DW, Zwiefelhofer D, Waite J, Perryman WL (2009). Distribution and movements of fin whales in the North Pacific Ocean. Mamm Rev.

[CR34] Moore SE, Stafford KM, Dahlheim ME, Fox CG, Braham HW, Polovina JJ, Bain DE (1998). Seasonal variation in reception of fin whale calls at five geographic areas in the North Pacific. Mar Mamm Sci.

[CR35] Moore SE, Waite JM, Mazzuca LL, Hobbs RC (2000). Mysticete whale abundance and observations of prey associations on the central Bering Sea shelf. J Cetacean Res Manage.

[CR36] Moore SE, Waite JM, Friday NA, Honkalehto T (2002). Cetacean distribution and relative abundance on the central eastern and southeastern Bering Sea shelf with reference to oceanographic domains. Prog Oceanogr.

[CR37] Moore SE, Stafford KM, Mellinger DK, Hildebrand JA (2006). Listening for large whales in the offshore waters of Alaska. Bioscience.

[CR38] Nieukirk SL, Stafford KM, Mellinger DK, Dziak RP, Fox CG (2004). Low-frequency whale and seismic airgun sounds recorded in the mid-Atlantic Ocean. J Acoust Soc Am.

[CR39] Oleson EM (2005) Calling behavior of blue and fin whales off California. PhD Dissertation, University of California San Diego, La Jolla, CA

[CR40] Oleson EM, Wiggins S, Hildebrand JA (2007). Temporal separation of blue whale call types on a southern California feeding ground. Anim Behav.

[CR41] Oleson EM, Calambokidis J, Burgess WC, McDonald MA, LeDuc CA, Hildebrand JA (2007). Behavioral context of Northeast Pacific blue whale call production. Mar Ecol Prog Ser.

[CR42] Ramcharitar J, Gannon DP, Popper AN (2011). Bioacoustics of fishes of the family Sciaenidae (Croakers and Drums). T Am Fish Soc.

[CR43] Rice DW (1998). Marine mammals of the world: systematics and distribution.

[CR44] Schevill WE, Watkins WA, Backus RH, Tavolga WN (1964). The 20-cycle signals and *Balaenoptera* (fin whales). Marine bio-acoustics.

[CR45] Širović A, Hildebrand JA, Wiggins SM, McDonald MA, Moore SE, Thiele D (2004). Seasonality of blue and fin whale calls and the influence of sea ice in the Western Antarctic Peninsula. Deep-Sea Res II.

[CR46] Širović A, Hildebrand JA, Thiele D (2006). Baleen whales in the Scotia Sea in January and February 2003. J Cetacean Res Manage.

[CR47] Širović A, Hildebrand JA, Wiggins SM (2007). Blue and fin whale call source levels and propagation range in the Southern Ocean. J Acoust Soc Am.

[CR48] Soldevilla MS, Wiggins SM, Hildebrand JA (2010). Spatial and temporal patterns of Risso’s dolphin echolocation in the Southern California Bight. J Acoust Soc Am.

[CR49] Stafford KM, Moore SE, Fox CG (2005). Diel variation in blue whale calls recorded in the eastern tropical Pacific. Anim Behav.

[CR50] Stafford KM, Mellinger DK, Moore SE, Fox CG (2007). Seasonal variability and detection range modeling of baleen whale calls in the Gulf of Alaska, 1999–2002. J Acoust Soc Am.

[CR51] Stafford KM, Citta JJ, Moore SE, Daher MA, George JE (2009). Environmental correlates of blue and fin whale call detections in the North Pacific Ocean from 1997 to 2002. Mar Ecol Prog Ser.

[CR52] Stirling I, Calvert W, Cleator H (1983). Underwater vocalizations as a tool for studying the distribution and relative abundance of wintering pinnipeds in the high Arctic. Arctic.

[CR53] Tershy BR (1992). Body size, diet, habitat use, and social behavior of Balaenoptera whales in the Gulf of California. J Mammal.

[CR54] Tershy BR (1993). Are fin whales resident to the Gulf of California?. Rev Invest Cient.

[CR55] Tershy BR, Breese D, Strong CS (1990). Abundance, seasonal distribution and population composition of balaenopterid whales in the Canal de Ballenas, Gulf of California, Mexico. Rep Int Whal Commn.

[CR56] Thomas JA, Demaster DP (1982). An acoustic technique for determining diurnal activities in leopard (*Hydrurga leptonyx*) and crab-eater (*Lobodon carcinophagus*) seal. Can J Zool.

[CR57] Thompson PO, Friedl WA (1982). A long term study of low frequency sounds from several species of whales off Oahu, Hawaii. Cetology.

[CR58] Thompson PO, Cummings WC, Ha SJ (1986). Sounds, source levels, and associated behavior of humpback whales, Southeast Alaska. J Acoust Soc Am.

[CR59] Thompson PO, Findley LT, Vidal O (1992). 20-Hz pulses and other vocalizations of fin whales, *Balaenoptera physalus*, in the Gulf of California, Mexico. J Acoust Soc Am.

[CR60] Víkingsson GA (1997). Feeding of fin whales (*Balaenoptera physalus*) off Iceland—diurnal and seasonal variation and possible rates. J Northw Atl Fish Sci.

[CR61] Watkins WA (1981). Activities and underwater sounds of fin whales. Sci Rep Whales Res Inst.

[CR62] Watkins WA, Schevill WE (1972). Sound source location by arrival-times on a non-rigid three-dimensional hydrophone array. Deep-Sea Res.

[CR63] Watkins WA, Tyack P, Moore KE, Bird JE (1987). The 20-Hz signal of finback whales (*Balaenoptera physalus*). J Acoust Soc Am.

[CR64] Watkins WA, Daher MA, Reppucci GM, George JE, Martin DL, DiMarzio NA, Gannon DP (2000). Seasonality and distribution of whale calls in the North Pacific. Oceanography.

[CR65] Wiggins SM, Hildebrand JA (2007) High-frequency Acoustic Recording Package (HARP) for broad-band, long-term marine mammal monitoring. In: International symposium on underwater technology 2007 and international workshop on scientific use of submarine cables and related technologies. Institute of Electrical and Electronics Engineers, Tokyo, Japan, pp 551–557

[CR66] Wiggins SM, Oleson EM, McDonald MA, Hildebrand JA (2005). Blue whale (*Balaenoptera musculus*) diel calling patterns offshore of Southern California. Aquat Mamm.

[CR67] Winn HE, Winn LK (1978). The song of humpback whale (*Megaptera novaeangliae*) in the West Indies. Mar Biol.

[CR68] Zar JH (1984). Biostatistical analysis.

